# Atrial fibrillation after cardiac surgery: to screen or not to screen?

**DOI:** 10.1093/cvr/cvaa352

**Published:** 2021-01-18

**Authors:** Barbara Casadei, Rohan Wijesurendra

**Affiliations:** Radcliffe Department of Medicine, Division of Cardiovascular Medicine, RDM, John Radcliffe Hospital, University of Oxford, UK

Atrial fibrillation (AF) is diagnosed in 20–40% of patients in the first week after cardiac surgery and is largely self-limiting, with the majority of patients being discharged from hospital in sinus rhythm (SR). However, that is not the end of it. The rate of subsequent AF development is higher in cardiac surgery patients who experience AF during hospital admission compared to those who do not^1^; patients with postoperative AF also have a longer hospital stay, a higher long-term risk of stroke (both ischaemic and haemorrhagic) and worse overall prognosis.^1–5^

Previous studies have defined post-operative AF as occurring between the surgical procedure and hospital discharge, and only systematically monitored heart rhythm during this time period. SEARCH-AF, a randomized trial of AF screening using a patch electrocardiogram (ECG) monitor, uncovered that an additional 20% of patients, who underwent cardiac surgery and had no in-hospital complications, develop new-onset AF early after hospital discharge meaning that, overall, the majority of patients in SR at the time of surgery may experience (mostly paroxysmal) AF in the first few months after the intervention. More specifically, SEARCH-AF randomized 336 cardiac surgery (mostly CABG) patients with no history of AF either pre- or post-operatively but high clinical stroke risk (defined as CHA_2_DS_2_-VASc ≥4, or CHA_2_DS_2_-VASc ≥2 with ≥1 of the following: chronic obstructive pulmonary disease (COPD) or sleep apnoea; eGFR <60 mL/min/1.73 m^2^; left atrial dilatation; body mass index (BMI) ≥30 kg/m^2^) to ECG monitoring (for 4 weeks) after hospital discharge vs. usual care (for the same period). AF was detected in 32 patients (19.6%) in the active arm (in almost all of them during the first 2 weeks of monitoring) compared with 3 (1.7%) in the ‘usual care’ arm. AF was generally short lasting, with a duration over 24 h occurring in only 5 of the monitored patients. This may have contributed to clinical decision-making regarding anticoagulation, which was commenced in only 13 out of 32 patients who were found to have AF in the active arm (vs. 8 in the control arm). This suggests that postoperative AF, particularly when detected by ECG monitoring rather than presenting with symptoms, is regarded as insufficient grounds for initiation of life-long anticoagulation, even in the presence of high clinical stroke risk.

Taking these findings into consideration, should AF screening be performed in all patients post-cardiac surgery (or in all patients after major surgery^2^) with a high clinical stroke risk?

SEARCH-AF was not designed or powered to establish whether AF screening confers any benefit to patients in terms of a reduction in major cardiovascular events. The finding that screening after hospital discharge identifies AF in as many as 20% of patients is interesting but rendered moot in terms of influencing patient management by the lack of compelling randomized evidence supporting anticoagulation in this clinical context, and by the (presumably related) reluctance to initiate it. Clearly, the more intensely one monitors cardiac rhythm the more AF one finds.^6^^,^^7^ But, as the title of the hotline session implies, ‘… and then what?’ Should all high-risk patients undergoing cardiac surgery be prescribed prophylactic antiarrhythmic treatment at hospital discharge? This question was partially answered by Gillinov *et al.*^8^ who randomized 523 hospitalized patients who developed one episode of AF lasting more than 60 min or multiple AF episodes after cardiac surgery to either a rate control approach or to rhythm-control therapy with amiodarone for 60 days. Duration of hospitalization (the primary outcome) did not differ between groups nor did mortality or serious adverse events. At 60 days, 93.8% of the patients in the rate control group and 97.9% of those in the rhythm-control group were in SR without AF for the previous 30 days (*P* = 0.02), and 84.2% and 86.9%, respectively, had been free from AF from discharge to 60 days (*P* = 0.41). There was a 25% rate of cross-over in both groups, which in the rhythm-controlled group was largely due to the toxic effects of amiodarone. Given that only six patients in total (four in the rate control and two in the rhythm-control group) experienced a cerebrovascular event during the 60-day follow-up, a much larger trial would be needed to evaluate the potential prognostic benefit of an aggressive rhythm-control strategy, which could include intra-operative pulmonary vein isolation and/or left atrial appendage removal or exclusion.

Should those who are found to have symptomatic or asymptomatic AF after cardiac surgery be started on oral anticoagulant therapy, if eligible according to the CHA_2_DS_2_-VASc score? The 10-year risk of all stroke (including haemorrhagic) in patients with postoperative AF diagnosed during their hospital admission in the Arterial Revascularization Trial (ART)^5^ was 6.3% (4.6–8.1%) vs. 3.7% (2.9–4.5%) in those in SR after surgery (∼20% of whom would still develop AF soon after hospital discharge, according to SEARCH-AF, implying that the relative risk of stroke associated with postoperative AF may be greater than that reported in this study).

In ART, exploratory analyses suggested that anticoagulation may be beneficial in those with postoperative AF and a CHA_2_DS_2_-VASc score of 4 or above who had a four-fold increase in the risk of cerebrovascular events on follow-up vs. the two-fold increase in risk observed in patients in post-operative SR with the same CHA_2_DS_2_-VASc score.^5^ In line with these findings and those from other non-randomized analyses, the 2019 ESC guidelines on the diagnosis and management of patients with AF state that long-term oral anticoagulation in patients at risk for stroke with post-operative AF after cardiac surgery ‘may be considered’ (Class IIb, Level of Evidence B), taking into account the anticipated net clinical benefit of anticoagulation and informed patient preferences.^9^ However, randomized evidence supporting this strategy is missing and consideration needs to be given to the fact that some patients who undergo CABG may be prescribed dual-antiplatelet therapy (DAPT) for 6–12 months.^10^ Whilst the addition of oral anticoagulation under these circumstances would greatly increase bleeding risk, it should be noted that the indication for DAPT after CABG in patients with stable coronary artery disease (CAD) (vs. those who present with an acute coronary syndrome) remains debated, with further clarity needed on the precise timing of initiation and duration of therapy.^10^ It is clear therefore that randomized trials investigating best management for the prevention of both thrombo-embolic events and graft occlusion (when appropriate) in patients undergoing cardiac surgery are sorely needed. A first step towards answers to some of these questions will be provided by PACES (Anticoagulation for new-onset post-operative atrial fibrillation after CABG; NCT04045665)—a multicentre, open-label, randomized trial comparing oral anticoagulation with vitamin K antagonist (international normalised ratio (INR) target 2–3) or any approved direct oral anticoagulant, in addition to antiplatelet therapy with aspirin 75–325 mg o.d. or a P2Y12-inhibitor (clopidogrel or ticagrelor), to aspirin 75–325 mg o.d. or a P2Y12-inhibitor in 3200 patients who developed new-onset postoperative AF (one episode >60 min or recurrent episodes) within 7 days of isolated CABG surgery. The primary effectiveness endpoint of this trial is a composite of death, stroke, transient ischaemic attack, myocardial infarction, systemic arterial, or venous thromboembolism at 90 days after randomization whereas the primary safety endpoint is Bleeding Academic Research Consortium Grade 3 or 5 bleeding. PACES is expected to complete in December 2023.

In summary, AF is a common complication of cardiac surgery, both shortly after the intervention and, as SEARCH-AF has just shown, post-hospital discharge. Post-operative AF is associated with longer hospital stay and a worse prognosis, specifically including an increase in both in-hospital and long-term stroke risk, particularly in those with a high (≥4) CHA_2_DS_2_-VASc score. However, the optimal management strategy for these patients remains unclear. Until large-scale randomized evidence is available to guide treatment, screening for AF after cardiac surgery is likely to be of little practical value.

**Conflict of interest:** none declared.

## Funding

We acknowledge funding from the British Heart Foundation and the NIHR Oxford Biomedical Research Centre.

## Figures and Tables

**Figure:**
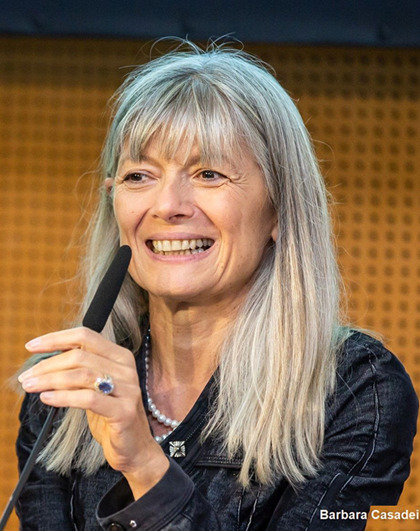


**Figure:**
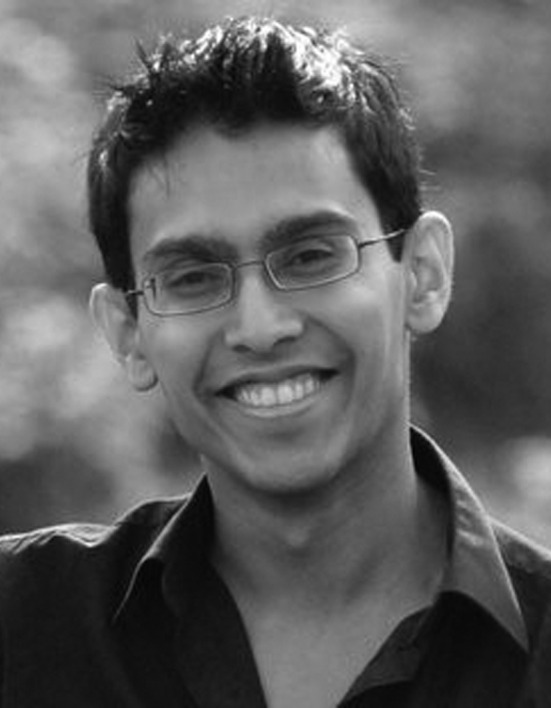


## Notes

From the AHA Scientific Sessions - To Screen or Not to Screen, and then What? Studies of Detection and Treatment of AF. Monday, Nov. 16, 2020.

## Authors

**Biography:** Barbara Casadei is a *British Heart Foundation* (BHF) Professor of Cardiovascular Medicine at the University of Oxford and Honorary Consultant Cardiologist at the Oxford University Hospitals NHS Trust where she leads the *Cardiovascular Theme* of the *National Institute for Health Research (NIHR) Biomedical Research Centre*. Professor Casadei graduated in Medicine (*cum Laude*) at the University of Pavia, Italy and moved to Oxford in 1989 to undertake her clinical and research training. She was awarded the *Joan and Richard Doll Fellowship* at Green College in 1991, a DPhil in Cardiovascular Medicine in 1995, and a *BHF Senior Research Fellowship* in 2001. She is a Fellow of the UK *Academy of Medical Sciences* and the Immediate Past President (2020–22) of the *European Society of Cardiology* (ESC). Her research programme spans from investigations in human tissue and cells to clinical trials with a focus on atrial fibrillation.

## Notes

**Biography:** Rohan Wijesurendra is a Clinical Lecturer in Cardiovascular Medicine at the University of Oxford and Specialty Registrar in Cardiology (Electrophysiology and Devices) at the Oxford University Hospitals NHS Trust. Dr Wijesurendra graduated in medicine from Cambridge University with several prizes and scholarships, and subsequently moved to Oxford to pursue postgraduate training and research. He was awarded a DPhil in Cardiovascular Medicine in 2018 for research exploring mechanisms underlying impaired left ventricular function in patients with atrial fibrillation using multi-parametric cardiac magnetic resonance; this work resulted in several high-impact publications and awards including the *Young Investigator Award in Clinical Science* of the *European Society of Cardiology* and the *Melvin Judkins Young Clinical Investigator Award* of the *American Heart Association*. His ongoing research interests include clinical trials and application of cardiac magnetic resonance in the field of cardiac rhythm management.
